# Draft Genome Sequences of Supercritical CO_2_-Tolerant Bacteria *Bacillus subterraneus* MITOT1 and *Bacillus cereus* MIT0214

**DOI:** 10.1128/genomeA.00140-15

**Published:** 2015-04-09

**Authors:** Kyle C. Peet, Janelle R. Thompson

**Affiliations:** Department of Civil and Environmental Engineering, Massachusetts Institute of Technology, Cambridge, Massachusetts, USA

## Abstract

We report draft genome sequences of *Bacillus subterraneus* MITOT1 and *Bacillus cereus* MIT0214 isolated through enrichment of samples from geologic sequestration sites in pressurized bioreactors containing a supercritical (sc) CO_2_ headspace. Their genome sequences expand the phylogenetic range of sequenced bacilli and allow characterization of molecular mechanisms of scCO_2_ tolerance.

## GENOME ANNOUNCEMENT

During geologic carbon sequestration (GCS), large quantities of CO_2_ are captured, compressed to supercritical (sc) state, and injected underground. Whether microbial activities transform injected CO_2_ is not well understood due to toxic effects of scCO_2_ (1–5). Samples from GCS sites at Otway Basin, Australia and Frio-2, Texas, were used as inocula for serial enrichment cultures in bioreactors containing scCO_2_, yielding strains *Bacillus subterraneus* MITOT1 and *Bacillus cereus* MIT0214, respectively (6). Tolerance of scCO_2_ was confirmed by growth of spores in pure cultures and was time and inocula density dependent. To investigate mechanisms of growth under scCO_2_, genomic DNA was sequenced.

MITOT1 was sequenced on the Illumina HiSeq 2000 platform (Beijing Genomics Institute). MIT0214 was sequenced on the Illumina GAIIx platform (MIT Biomicrocenter). Paired-end 100 bp reads were quality trimmed (removing 10 starting and 20 trailing bases) and assembled *de novo* with CLC Genomic Workbench with automatic k-mer sizes of 23 and 21, yielding 185 and 238 contigs of >500 bp, respectively. The draft genome of MITOT1 is 3.9 Mbp with 42.1% G+C content, while the MIT0214 draft genome is 5.6 Mbp with 34.9% G+C content. Annotation using the RAST server (7) predicted 4,021 (with 1,235 hypothetical) and 5,640 (with 1,399 hypothetical) coding sequences in MITOT1 and MIT0214.

Phylogenetic analysis of the 16S rRNA gene placed MITOT1 within a clade of bacilli isolated from diverse environments including deep subsurface, soil, manufacturing effluent, and fermented seafood (8–12), some of which are capable of anaerobic reduction of Fe(III), Mn(IV), Se(VI), and As(V) (8, 10). The closest relative by BLASTn of the 16S rRNA gene was *B. subterraneus* HWG-A11 (98.6% identity). The nearest genome sequenced strain (98.1% 16S rRNA identity) was *B. boroniphilus* DSM17376, isolated from boron-contaminated soil (13) and sharing 83.3% average nucleotide identity (ANI) (14) with 2,600 sequence homologs (>60% identity). RAST functional comparison of the MITOT1 and *B. boroniphilus* DSM17376 genomes with closely related bacilli (strain 1NLA3E, *B. infantis* NRRL B-14911, *B. megaterium* DSM319, and *B. coagulans* 36D1) predicted multiple anaerobic respiratory reductases and terminal cytochrome C oxidases unique to MITOT1 and *B. boroniphilus*, pointing to diverse catabolic potential for this group (15, 16).

Strain MIT0214 was most similar to *B. cereus* ATCC 14579 by BLASTn of 16S rRNA (99.8% identity), sharing 98.5% ANI and 4,858 sequence homologs (>60% identity). *B. cereus* strains have been isolated from diverse environments, including strain Q1 (92.5% ANI; 4,617 sequence homologs) from an oil reservoir (17). Comparisons among genomes of MITOT1, MIT0214, and the closely related sequenced genomes did not reveal clear signatures associated with scCO_2_ tolerance, which is unsurprising in light of recent observations that tolerance is widespread among bacilli (6). Availability of draft genome sequences for *B. subterraneus* MITOT1 and *B. cereus* MIT0214 from two GCS sites will facilitate future work targeting gene/protein expression to advance mechanistic insights into scCO_2_ tolerance.

### Nucleotide sequence accession numbers.

This whole-genome shotgun project has been deposited at DDBJ/EMBL/GenBank under the accession numbers JXIQ00000000 and JXDH00000000. The versions described in this paper are the first versions.
